# Simultaneous, Multi-Channel, Near-Infrared Fluorescence Visualization of Mesenteric Lymph Nodes Using Indocyanine Green and Methylene Blue: A Demonstration in a Porcine Model

**DOI:** 10.3390/diagnostics13081469

**Published:** 2023-04-18

**Authors:** Nariaki Okamoto, Zaid Al-Difaie, Max H. M. C. Scheepers, Danique J. I. Heuvelings, María Rita Rodríguez-Luna, Jacques Marescaux, Michele Diana, Laurents P. S. Stassen, Nicole D. Bouvy, Mahdi Al-Taher

**Affiliations:** 1 IRCAD, Research Institute against Digestive Cancer, 67091 Strasbourg, France; 2 ICube Laboratory, Photonics Instrumentation for Health, 67081 Strasbourg, France; 3 GROW School for Oncology and Developmental Biology, Maastricht University, 6229 ER Maastricht, The Netherlands; 4 Department of Surgery, Maastricht University Medical Center, 6229 ER Maastricht, The Netherlands; 5 NUTRIM School of Nutrition and Translational Research in Metabolism, Maastricht University, 6229 HX Maastricht, The Netherlands

**Keywords:** colorectal surgery, image-guided surgery, optical imaging, simultaneous, multi-channel, near-infrared fluorescence image, lymph node, indocyanine green, methylene blue

## Abstract

Near-infrared fluorescence (NIRF) image-guided surgery is a useful tool that can help reduce perioperative complications and improve tissue recognition. Indocyanine green (ICG) dye is the most frequently used in clinical studies. ICG NIRF imaging has been used for lymph node identification. However, there are still many challenges in lymph node identification by ICG. There is increasing evidence that methylene blue (MB), another clinically applicable fluorescent dye, can also be useful in the intraoperative fluorescence-guided identification of structures and tissues. We hypothesized that MB NIRF imaging could be used for lymph node identification. The aim of this study was to evaluate the feasibility of intraoperative lymph node fluorescence detection using intravenously (IV) administered MB and compare it to ICG via a camera that has two dedicated near-infrared (NIR) channels. Three pigs were used in this study. ICG (0.2 mg/kg) was administered via a peripheral venous catheter followed by immediate administration of MB (0.25 mg/kg). NIRF images were acquired as video recordings at different time points (every 10 min) over an hour using the QUEST SPECTRUM^®^ 3 system (Quest Medical Imaging, Middenmeer, The Netherlands), which has two dedicated NIR channels for simultaneous intraoperative fluorescence guidance. The 800 nm channel was used to capture ICG fluorescence and the 700 nm channel was used for MB. The target (lymph nodes and small bowel) and the background (vessels-free field of the mesentery) were highlighted as the regions of interest (ROIs), and corresponding fluorescence intensities (FI) from these ROIs were measured. The target-to-background ratio (TBR) was then computed as the mean FI of the target minus the mean FI of the background divided by the mean FI of the background. In all included animals, a clear identification of lymph nodes was achieved at all time points. The mean TBR of ICG in lymph nodes and small bowel was 4.57 ± 1.00 and 4.37 ± 1.70, respectively for the overall experimental time. Regarding MB, the mean TBR in lymph nodes and small bowel was 4.60 ± 0.92 and 3.27 ± 0.62, respectively. The Mann-Whitney U test of the lymph node TBR/small bowel TBR showed that the TBR ratio of MB was statistically significantly higher than ICG. The fluorescence optical imaging technology used allows for double-wavelength assessment. This feasibility study proves that lymph nodes can be discriminated using two different fluorophores (MB and ICG) with different wavelengths. The results suggest that MB has a promising potential to be used to detect lymphatic tissue during image-guided surgery. Further preclinical trials are needed before clinical translation.

## 1. Introduction

Colorectal cancer (CRC) is the third leading cause of cancer and the second leading cause of cancer-related mortality worldwide. It is estimated that there were approximately 1 million cases of colorectal cancer and 88,000 deaths worldwide in 2018 [1]. The surgical approach to colorectal cancer has changed significantly over the last few decades, including the introduction of total mesorectal excision (TME) and complete mesocolic excision (CME), advances in minimally invasive procedures, preoperative chemoradiotherapy, and surgical optical imaging technologies. 

Over the past few decades, one of the most significant innovations in the history of colorectal cancer treatment has been total mesorectal excision (TME) proposed by Heald et al. in 1982 [2]. Due to the embryologic development, the rectum and mesorectum are considered part of a single anatomical structure which surrounded by the visceral pelvic fascia. This thorough understanding of anatomical features allow TME to provide a safe “en bloc” resection of the rectum and its mesorectum, which include the majority of regional lymph nodes, lymphatic vessels, and surrounding fat tissue [3]. The TME “en bloc” resection was pivotal in significantly reducing the local recurrence rate after rectal cancer surgery, decreasing the incidence of permanent stomas, improving the curative resection rate, and improving survival and tumor-free survival rates. TME has become the “gold standard” surgical treatment for rectal cancer worldwide [4,5].

CME proposed by Hohenberger et al. consists of resecting the tumor-bearing bowel segments with “en bloc” resection of the mesocolon along its embryonic fascial planes based on the concept of TME [6,7]. CME, which is characterized by a central vascular ligation and a larger mesenteric specimen, results in a greater number of lymph nodes than non-CME resections. This superior lymphadenectomy may account for the lower reported risk of local recurrence in some studies [6,7,8,9]. However, several articles have shown that wider excisions did not improve oncologic benefits and CME has been associated with increasing concerns regarding the elevated morbidity and risk of postoperative complications such as injuries to the splenic and superior mesenteric veins [10,11,12,13,14].

In oncological surgery, accurate lymph node assessment enables pathological staging and prognosis-guiding postoperative therapies. Importantly, adequate lymphadenectomy has shown to improve overall survival [15]. In colorectal cancer, the main goal of oncologic resection is resecting the primary tumor along with its blood supply and lymph node drainage [6,16,17,18,19,20]. Numerous studies have been conducted on surgical imaging technologies, in digestive surgery, near-infrared fluorescence (NIRF) image-guided surgery has been studied extensively to understand its influence in surgical outcomes, such as the prevention of complications, diagnosis, staging, and improved radical resection of cancer all over the world [21]. NIRF imaging is a relatively new technology that uses a near-infrared light, allowing surgeons to see beyond the normal white light spectrum, helping them visualize not only the surface of the structure but also the inside of the structure [22]. It requires a locally or systemically administration of a fluorescent dye and an optical imaging system able to excite, detects, and measures the fluorescent dye spectra.

Indocyanine green (ICG), the most frequently used clinically approved fluorescence dye, absorbs light at 790 to 805 nm and has a peak emission at 835 nm when it is bound to α1 lipoprotein in the body [23]. Since ICG was approved by Food and Drug Administration (FDA) in 1959, it has been used primarily for the evaluation of blood vessels in contrast studies such as blood flow assessment in the retina [24], and since then, its field of applications have broadly expanded. ICG has a half-life of 150 to 180 s, and it is removed from circulation rapidly and exclusively by the liver to bile juice. Thanks to the hepatic clearance, ICG has been used for the assessment of liver function, measurement of blood flow in the liver, and extrahepatic bile duct differentiation [16].

ICG is a non-toxic dye and the reported probability of allergy is very low (1:40,000–1:60,000 cases) [25,26,27,28]. Intravenous administration of ICG has been used in, amongst others, cardiac output assessment [23], intraoperative tumor demarcation [29], and perfusion assessment at the level of the anastomosis [30,31,32,33,34] as a fluorescent dye in colorectal surgery. Since it is known that ICG has a tendency to migrate to lymphatic tissues, ICG NIRF imaging has been used for sentinel node identification [35,36,37], and intraoperative real-time lymphatic flow visualization [15,38]. However, one of the main drawbacks of ICG is its high affinity for albumin and progressive diffusion in tissues, which reduces its capacity to accurately locate affected areas [39].

Methylene blue (MB) is another clinically approved dye and has a peak excitation wavelength of ~700 nm and an emission wavelength of 688 nm [40,41]. Due to its renal clearance, NIRF imaging with MB has mostly been used in colorectal cancer surgery for intraoperative visualization of the urinary tract [41,42,43]. Moreover, there have been several studies using MB to detect sentinel lymph nodes in colorectal cancer [44] and for postoperative lymph node harvesting [45,46]. Its use is limited in a few optical imaging devices since most NIRF imaging systems are designed for imaging with ICG and dyes with smiliar optical properties. An example of a dual imaging system is the QUEST SPECTRUM^®^ 3 (Quest Medical Imaging, Middenmeer, The Netherlands) camera which has two dedicated near-infrared (NIR) channels for simultaneous intraoperative fluorescence guidance, which thus enables imaging of both MB and ICG. As previously stated, the differences in peak excitation and pharmacodynamics between MB and ICG reflect their different principal applications in the clinical setting. In colorectal surgery, Polom W et al. were able to identify the location of the ureter using MB while assessing intestinal perfusion to determine the optimal anastomotic site with ICG using a single camera system. Their results highlight the feasibility of multi-wave imaging to aid in preventing two of the most common complications in colorectal surgery, namely anastomotic leak, and ureteral injury. The relevance of simultaneous, multi-channel, near-infrared fluorescence imaging may represent a path toward increased surgical safety [47].

This study’s primary aim was to validate whether intravenously administered MB and ICG act simultaneously as fluorescence dyes for lymph node detection. As a secondary aim, we compared the fluorescence performance of MB and ICG in lymph node detection.

The in-vivo preclinical evaluation of intravenous (IV) injected MB and ICG simultaneously shows feasibility in detecting lymph nodes during digestive surgery. We discuss relevant results including the faster washout of MB as well as less background fluorescence providing easier detection of lymph nodes.

## 2. Materials and Methods

This study was performed at the animal laboratory of the Maastricht University Medical Center (MUMC+, Maastricht, The Netherlands). The experiments were performed in three female Dutch landrace pigs (mean weight: 40 kg). Animals were used in compliance with Dutch regulations and legislation concerning animal research, and the study was performed according to a protocol approved by the Experimental Animal Committee of Maastricht University (DEC-UM) (approval code: 2017-021-001). All surgical procedures were performed under general anesthesia. Intramuscular injection of azaperone 3 mg/kg, ketamine 10 mg/kg, atropine 0.05 mg/kg, thiopental 10–15 mg/kg, isoflurane (dose depending on the effect), and oxygen 20–40 mL/kg/min was used as a standard medication to ensure appropriate sedation and analgesia. All variations in vital parameters were continuously monitored. At the end of the protocol, animals were euthanized with a lethal dose of pentobarbital (40 mg/Kg).

### 2.1. Fluorescence Imaging System

The QUEST SPECTRUM^®^ 3 system was developed for NIRF image-guided surgery in an open surgical setting. This fluorescence imaging system has two dedicated NIR channels in the electromagnetic spectrum (700 and 800 nm), which allows for simultaneously visualization of ICG and MB. The channels were changed directly on the monitor touchscreen by simply clicking on the preferred channel. Screen settings included the Red/Green/Blue image, NIRF response in grayscale image, overlay of fluorescent image in green with the color image, and overlay of the fluorescent response image in Color (Blue to Red) on the Color image in grayscale.

### 2.2. Preparation of the Dyes

ICG (Verdye, Diagnostic Green GmbH, Aschheim, Germany) was diluted in a sterile H_2_O solution to a concentration of 2.5 mg/mL and was injected intravenously at a concentration of 0.2 mg/kg body weight via a peripheral venous line. The dose is based on the findings of our previous publication in which this was the most frequently used dose in clinical settings [8].

MB (Provepharm Life Solutions, Marseille, France) was diluted in a sterile phosphate-buffered saline solution to a concentration of 1 mg/mL and was injected intravenously at a concentration of 0.25 mg/kg body immediately following the injection of ICG. This dosage was determined based on previous literature [42].

### 2.3. Surgical Procedure

**Figure 1:**
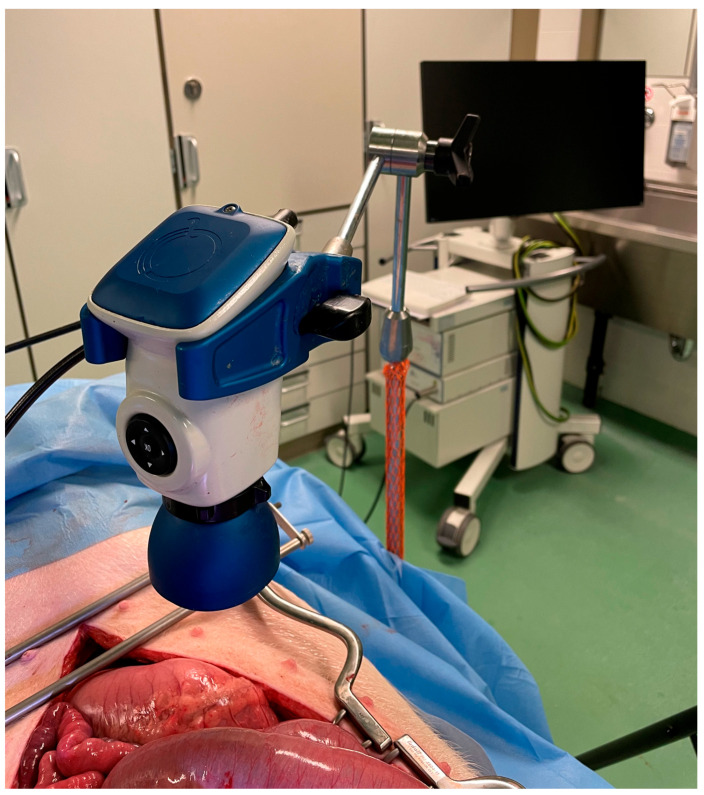
Set-up during experiments: The abdominal cavity was accessed via a midline laparotomy to expose the regions of interest (i.e., small bowel and mesenteric lymph nodes). The distal lens of the QUEST camera^®^ 3 (Quest Medical Imaging, Middenmeer, The Netherlands) was positioned 15 cm above the regions of interest. External light interference was avoided during the acquisition of images.

To expose the ROI mesenteric lymph nodes and small bowel, the abdominal cavity was accessed via a midline laparotomy, and a self-retaining abdominal wall retractor was placed. The camera tip was positioned at 15 cm above the ROI. During NIRF imaging, environmental lights were turned off preventing ambient light interference, and the average capture time was 20 s per image. The experimental surgical setting is represented in Figure 1.

### 2.4. Image Acquisition and Statistical Analysis

NIRF images were acquired from video recording after 1, 10, 20, 30, 40, 50, and 60 min of dye administration, respectively, using the QUEST SPECTRUM^®^ 3.

First, the target (mesenteric lymph nodes and small bowel) and the background (vessels-free field of the mesentery) were highlighted as ROI, and corresponding fluorescence intensities (FI) which were expressed in arbitrary units (a.u.) from these ROIs were measured. Fluorescence intensity measurement was performed using the Quest Artemis (Quest Medical Imaging, Middenmeer, The Netherlands) software (TBR tool v1.0.). The target-to-background ratio (TBR) was then computed as the mean FI of the target divided by the mean FI of the background.

To identify any potential superiority in terms of dye performance, ratios were used dividing lymph node TBR/small bowel TBR per dye. A ratio comparison of MB and ICG was made using a Mann-Whitney U test for continuous variables. A two-tailed analysis with a *p* value < 0.05 was considered statistically significant. These analyses were performed using commercially available database software (Excel version 2021, Microsoft Corporation, Redmond, WA, USA).

## 3. Results

**Figure 2:**
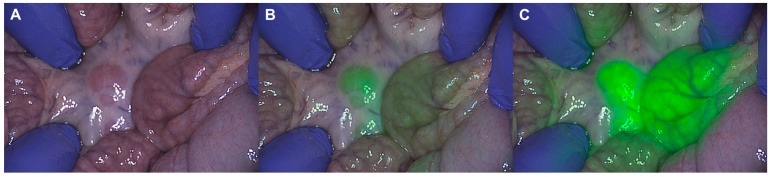
Picture of mesenteric lymph nodes and bowel after 20 min of intravenous MB and ICG administrations. (**A**) The RGB image. (**B**) Overlay fluorescence picture at 700 nm (fluorescence for MB). (**C**) Overlay fluorescence picture at 800 nm (fluorescence for ICG). MB, methylene blue; ICG, indocyanine green.

**Figure 3:**
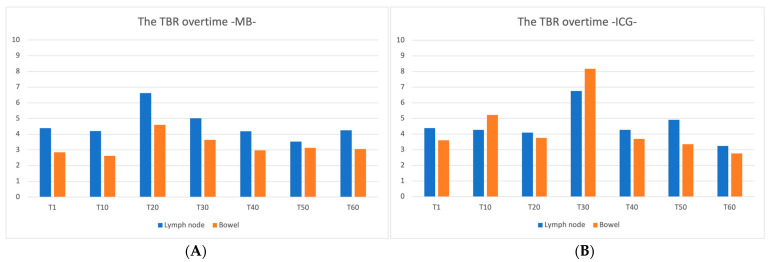
The TBR over time, (**A**) MB (**B**) ICG. TBR, target-to-background ratio; MB, methylene blue; ICG, indocyanine green.

In all three animals included, it was possible to clearly visualize the lymph nodes under NIRF imaging using either of the dyes administered during the overall surgical procedure (Figure 2). Subjective evaluation of lymph node visualization with two dyes by two experienced surgeons was performed on a four-point scale (Excellent/Good/Average/Poor), with Good or Excellent at all intraoperative time points. The TBR for each time point is represented in Figure 3. The TBRs of MB and ICG were greater than 2.0 at all measured time points. The mean TBR of ICG in lymph nodes and small bowel was 4.57 ± 1.00 and 4.37 ± 1.70, respectively, for the overall experimental time. Regarding MB, the mean TBR in lymph nodes and small bowel was 4.60 ± 0.92 and 3.27 ± 0.62, respectively. The Mann-Whitney U test of lymph node TBR/small bowel TBR comparing ICG to MB showed that the value for MB was statistically significantly higher (*p* = 0.012). No complications related to dye administrations occurred.

## 4. Discussion

In this study, we validated that the two dyes (MB and ICG) can be used in parallel in an animal model for the identification of mesenteric lymph nodes. After simultaneous intravenous administration of the dyes, the NIRF imaging of lymph nodes and bowel was succesfull in both imaging modalities. The TBR of MB in mesenteric lymph nodes was not inferior to that of ICG. A possible explanation for these results is that there was a lower MB FI in the small bowel in comparison to ICG FI which was higher in the intestines. ICG possesses a higher FI background even in the vessels-free field of the mesentery. Additionally, since MB has a shorter washout time from the bowel than ICG, it may offer an enhanced potential for mesenteric lymph node detection as the FI background decreases more rapidly than when using ICG. 

In CRC, lymph node metastasis modifies staging, and the stage determines whether postoperative adjuvant chemotherapy is indicated. Results of a secondary analysis of the Intergroup trial INT-0089 showed that an increased number of lymph nodes retrieved correlates with a prolonged survival rate in both negative and positive cases of lymph node metastasis [48]. Swanson et al. showed that the number of lymph nodes examined, even in T3N0 colon cancer without preoperative lymph node metastasis, linearly correlated with 5-year survival [49]. International guidelines recommend the evaluation of 12 or more lymph nodes [50,51,52], However, scientific evidence is still lacking and it is still unclear if there is an absolute number that is considered as a gold standard for routine analysis [53]. Goldstein stated that a minimum of 30 lymph nodes in a hypothetical specimen was required to reach a probability of 80% to identify a single metastasis [54]. In other words, intraoperative lymph node detection and resection represent an essential component in predicting the long-term prognosis of CRC and in determining postoperative treatment strategies.

A systematic review and meta-analysis by Emile et al. showed that studies on fluorescent lymph node imaging differ with respect to ICG concentration, dose, injection site (submucosal, subserosal, combined submucosal and subserosal, intravenous), and timing of injection (preoperative, intraoperative, both preoperative and intraoperative) [55].

For fluorescence lymph node detection and lymphatic mapping using ICG, the dye is often injected in the subserosal or the submucosal layers where the main lymphatic network is located around the cancerous tissue [38,55,56]. However, this technique requires high technical precision during injection to prevent dye spillage, since abdominal cavity leakage alters the surgical field with false-positive fluorescence regions [57]. Regarding technical details during laparoscopic subserosal ICG injection, the lack of tactile feedback could cause the needle tip to reach the muscle layer, hence injecting the wrong layer resulting in inadequate dye absorption into the lymphatics [55]. Nishigori et al. studied 21 patients who underwent laparoscopic CRC surgery to evaluate both lymphatic and blood flow with ICG fluorescence imaging. To evaluate lymph flow, authors injected ICG into the submucosa around the tumor under colonoscopic guidance, with a timing of injection comprised either between 1 and 3 days before surgery or intraoperatively. To evaluate blood flow, ICG was injected intravenously during the procedure. Inadequate lymphatic flow delineation resulted in non-detection in three cases. In the two cases involving intraoperative IV injection, ICG leakage was a problem in both [15].

**Figure 4:**
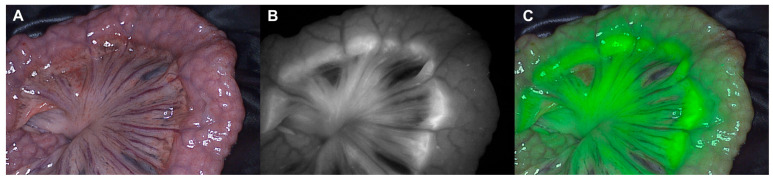
Picture of ascites after 40 min of IV ICG administration. (**A**) The RGB image. (**B**) Fluorescence picture at 800 nm (**C**) Overlay fluorescence picture at 800 nm.

**Figure 5:**
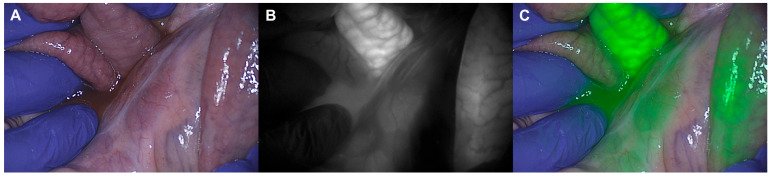
Picture of mesenteric edema after 40 min of IV ICG administration. (**A**) The RGB image. (**B**) Fluorescence picture at 800 nm (**C**) Overlay fluorescence picture at 800 nm.

A study by Liberale et al. reported that intraoperative IV administration of ICG for lymph node detection was feasible. Authors showed that intraoperative and postoperative fluorescence imaging might be a new tool to detect metastatic lymph nodes in patients with CRC, allowing for a more accurate staging [58]. In the previously quoted systematic review, the IV administration of ICG showed the second highest sensitivity, specificity, and accuracy for lymph node detection behind the combined subserosal and submucosal injection [59]. However, pooled results were based on only two lymph node mapping studies using IV ICG. At present, the preferred method of administration and dosage of ICG for fluorescence lymph node mapping is still under debate, and further clinical trials are highly necessary to establish the best reliable technique. ICG also diffuses into ascites and edema because of its albumin binding. In this experiment, some animals developed a degree of ascites or mesenteric edema after laparotomy. In those that did, ICG fluorescence images showed comparable FI in edema ROIs to that of the lymph nodes (Figure 4). Ascites that increased over time during surgery also showed similar FI. (Figure 5). As a result, the ICG fluorescence present in either ascites or edema may reduce the sensitivity of mesenteric lymph node detection during NIRF-guided surgery.

MB is hydrophobic and does not bind to albumin as opposed to ICG. Jun Li et al. reported NIRF imaging using MB for the identification of lymphatic vessels, sentinel lymph node location, and breast lymphatic flow patterns in breast cancer. They reported that MB causes less background fluorescence contamination than ICG, and MB could be suitable for observation due to its higher absorption and to the fact that it can be clearly detected across the skin and adipose tissue [59].

The fluorescence of MB injected simultaneously with ICG could be used to confirm whether the fluorescent ROIs are either mesenteric lymph nodes or mesenteric edema. The intravenous injection of two dyes may increase the sensitivity of mesenteric lymph node detection, thereby avoiding complex procedures such as subserosal and submucosal injections. In addition, this may prevent any excessive mesenteric injury or inadequate lymph node resection that could occur when ICG is administered alone.

Additionally, MB in the lymphatic network and lymph nodes stains a blue color in the specimen. The number of detectable lymph nodes depends on age, gender, type of malignancy, and tumor location [60]. Besides, preoperative adjuvant chemoradiation has become the standard of care for advanced rectal cancer in recent years. Neoadjuvant chemoradiotherapy reduces lymph node size, which causes a decrease in lymph node harvesting [61,62,63]. Märkl et al. reported a study in which MB was injected into the superior rectal artery ex vivo, originally performed to evaluate the accuracy of TME [64]. Other authors found that injecting MB postoperatively directly into the vessels of rectal resection specimens improved the lymph node detection rate [45]. Although it must be mentioned that this is an arterial injection and the concentration administered is high, they have shown that MB transferred from the blood vessels into the lymphatic flow stains the lymph nodes. Several studies have shown that higher doses of MB improved postoperative lymph node harvesting from rectal cancer specimens [45,46].

Another potential application of the presented imaging set-up, apart from the tested lymph node and bowel detection, is to assess blood flow and ureteral recognition as these are two other properties of the ICG and MB dyes. Ureteral injury is a serious complication during intrapelvic surgery, and the majority of iatrogenic ureteral injuries may lead to severe morbidity. The incidence of ureteral injury in colorectal surgery is not that high (0.25–1.1%). However, several studies showed that only 15% to 30% of intraoperative ureteral injuries are recognized. It most frequently occurs during proctectomy and sigmoidectomy colorectal cancer surgery [65]. Andersen et al. reviewed 18,474 colorectal resections of the Danish National Colorectal Cancer database and reported that laparoscopic procedures correlate with the risk of iatrogenic ureteral injury [66]. The surgeon should be familiar with the anatomy of the ureter and the risk factors for injury [67]. Visualization of the ureter is very important, especially after reoperation or radiation therapy. Intraoperative ureteral identification also contributes to the education of young surgeons in anatomy. As referred to earlier, ICG is cleared via the bile juice. The fluorescent urinary tract visualization with ICG requires the insertion of one or several ureteral catheter(s) via cystoscopy requiring an additional invasive procedure which may increase the risk of perioperative complications [68]. Several studies have been published on ureteral fluorescence using preclinical fluorophores with emission peaks similar to ICG. Since these novel fluorophores (including IRDye^®^ 800CW, IRDye^®^ 800BK, IRDye^®^ 800NOS [69], and IS-001 [70]) contribute to urinary clearance, they have shown promising results in detecting the ureter. However, further clinical translation is necessary since these dyes are currently not available in the marketplace.

As a result, since MB is excreted through the urine, it may represent the best way to intraoperatively identify ureters during colorectal surgery. Several studies have been published in which MB is injected intravenously and the ureter is visualized with fluorescence [41,42,43]. The first study of NIRF visualization of the ureter using MB was proposed by Verbeek et al. in 12 patients. In this study, the authors showed correct visualization of both ureters after intravenous injection of MB [43]. Later on, Polom et al. published a study on simultaneous fluorescent visualization of ureter and bowel perfusion via the administration of MB and ICG respectively using the laparoscopic camera system that was used in our study. The fluorescent visualization of the ureter was successful in 11 out of 12 patients (96.1%), and in all patients, bowel perfusion by means of ICG was successfully confirmed [47]. As a result, simultaneous assessment using MB and ICG is feasible and represent a comprehensive NIR guidance, helping surgeons to detect lymph nodes, to reduce the risk of anastomotic leakage by assessing local tissue perfusion, and to reduce the risk of complications by detecting ureters.

However, there are some drawbacks regarding the administration of MB that should be discussed. MB is contraindicated in patients with glucose-6-phosphate dehydrogenase (G6PD) deficiency and Heinz body anemia. MB is a safe agent when used at therapeutic doses of 2mg/kg or less [71]. In high doses or in patients with renal failure, MB may induce severe adverse effects, such as coronary vasoconstriction, arrhythmias, and hemolytic anemia [71]. While it is prudent to use the lowest dose of MB necessary for a given case to minimize the potential for toxic manifestations [72], several studies of fluorescence ureteral visualization with the administration of MB showed that higher doses of MB resulted in better ureteral visualization [42,43]. MB administration may cause false elevations in methemoglobin levels with CO-oximetry and pulse oximetry [73]. Finally, excess doses of MB may paradoxically increase oxidant stress and methemoglobinemia [74]. During this experiment, we did not observe any such changes in vital signs after MB administration.

Further experiments are necessary to determine the optimal method of administration, timing, and dosage of MB dye, particularly for lymph node mapping based on these previous studies.

The limitation of the present study lies in the fact that it is a small-number pilot study using a porcine model. The mesentery of an adult human is not as thin as that of a pig. The mesenteric lymph nodes of humans, which are usually embedded in adipose tissue, are generally more difficult to detect, an therfore, the current results should be interpreted with causion to predict whether this method can be generalized to humans. Additionally, the current study and the imaging system were used in an open surgery setting, and laparotomy was required for image acquisition while in clinical setting, minimally invasive laparoscopic surgery is the standard treatment in CRC surgery. At present, a laparoscopic camera equipped with a double-wavelength imaging system is available, enabling further research to establish the accuracy of this imaging system in minimally invasive surgery settings.

## 5. Conclusions

This feasibility study proves that mesenteric lymph nodes can be discriminated using two different fluorophores (MB and ICG) at different wavelengths. Based on superior discrimination from the background, MB has a promising potential to be used to detect lymphatic tissue during image-guided surgery. Further preclinical trials are needed before clinical translation.

## Data Availability

The datasets generated or analyzed during the current study are included in this published article.
